# Evidence for Mitochondrial Genome Methylation in the Yeast *Candida albicans*: A Potential Novel Epigenetic Mechanism Affecting Adaptation and Pathogenicity?

**DOI:** 10.3389/fgene.2018.00166

**Published:** 2018-05-29

**Authors:** Thais F. Bartelli, Danielle C. F. Bruno, Marcelo R. S. Briones

**Affiliations:** ^1^Laboratory of Evolutionary Genomics and Biocomplexity, Department of Microbiology, Immunology and Parasitology, Federal University of São Paulo, São Paulo, Brazil; ^2^Laboratory of Medical Genomics, A. C. Camargo Cancer Center, São Paulo, Brazil; ^3^Department of Health Informatics, Federal University of São Paulo, São Paulo, Brazil

**Keywords:** *Candida albicans*, mitochondrial genome methylation, mtDNA, hypoxia, heat shock

## Abstract

The commensal yeast *Candida albicans* is an opportunistic pathogen. In order to successfully colonize or infect the human body, the fungus must adapt to the host’s environmental conditions, such as low oxygen tension (hypoxia), temperature (37°C), and the different carbon sources available. Previous studies demonstrated the adaptive importance of *C. albicans* genetic variability for its pathogenicity, although the contributions of epigenetic and the influence of environmental factors are not fully understood. Mitochondria play important roles in fungal energetic metabolism, regulation of nuclear epigenetic mechanisms and pathogenicity. However, the specific impact of inter-strain mitochondrial genome variability and mitochondrial epigenetics in pathogenicity is unclear. Here, we draw attention to this relevant organelle and its potential role in *C. albicans* pathogenicity and provide preliminary evidence, for the first time, for methylation of the yeast mitochondrial genome. Our results indicate that environmental conditions, such as continuous exposure for 12 weeks to hypoxia and 37°C, decrease the mitochondrial genome methylation in strains SC5314 and L757. However, the methylation decrease is quantitatively different in specific genome positions when strains SC5314 and L757 are compared. We hypothesize that this phenomenon can be promising for future research to understand how physical factors of the host affect the *C. albicans* mitochondrial genome and its possible impact on adaptation and pathogenicity.

The yeast *Candida albicans* is responsible for severe mucosal and bloodstream opportunistic infections, with nearly 400,000 nosocomial cases worldwide and high mortality rates (46–75%) (Brown et al., 2012). In normal conditions this fungus is a harmless human commensal, successfully colonizing diverse niches, such as skin, urogenital and gastrointestinal tracts (Brock, 2009; Huffnagle and Noverr, 2013; Underhill and Iliev, 2014). The metabolic plasticity of *C. albicans* allows its survival and response to multiple environmental stimuli simultaneously, such as temperature, oxygen, and nutrient availability (Brown et al., 2014). The remarkable genetic variability among *C. albicans* isolates suggests that this opportunistic pathogen undergoes adaptation and microevolution when colonizing the human body (Morschhäuser et al., 2000; Forche et al., 2009; Wartenberg et al., 2014). Although intraspecific variation in the nuclear DNA is well-established, the evolutionary rate of *C. albicans* mitochondrial DNA (mtDNA) and its effect on its fitness and virulence is not clear.

Besides genetic diversity, several studies address the distribution and significance of *C. albicans* epigenetic plasticity as an adaptive mechanism (Zordan et al., 2006; Lopes da Rosa and Kaufman, 2012; Tscherner et al., 2015; Freire-Benéitez et al., 2016). Epigenetic factors, such as DNA methylation, can regulate the cellular response in absence of sequence changes in the DNA, and these factors can be modified and reversed by internal and external cellular stimuli, such as pollutants, oxidative stress, temperature, and nutrients (Byun and Baccarelli, 2014). Currently the only data available on *C. albicans* epigenetic regulation focus on its nuclear genome, mainly on modulation of morphology and other virulence factors, such as white-opaque switching (Zordan et al., 2006; Mishra et al., 2011; Zhang et al., 2013; Kim et al., 2015; Tscherner et al., 2015; Freire-Benéitez et al., 2016). Mapping of *C. albicans* nuclear genome hypermethylated sites identified genes involved in morphogenesis and hyphal growth (16.7%), white-opaque switching (3.3%), iron use (6.7%), drugs resistance and signaling (12%), stress response (7.3%), and genes involved in regulatory activities such as chromatin organization (3.3%), cycle or cell division (7.3%), biogenesis and protein transport (12.7%), DNA/RNA processing (5.3%), pathogenesis or virulence (2%), and carbohydrate metabolism (1.3%). It was also observed that, in this species, methylation occurs at both CpG and CH sites (H = Adenine, Cytosine, or Thymine), mainly in the gene bodies, instead of the promoters (Mishra et al., 2011).

In mammalian mitochondria the DNA methyltransferase enzymes DNMT1 (Shock et al., 2011) and DNMT3A (Chestnut et al., 2011) are responsible for DNA methylation and the enzymes TET1 and TET2 (10–11 translocation) (Dzitoyeva et al., 2012; Bellizzi et al., 2013) catalyze demethylation by converting 5methylcytosines (5mC) into 5-hydroxymethylcytosines (5hmC), which are passively eliminated during DNA replication or actively reversed in cytosines by iterative oxidation followed by base excision repair (Kohli and Zhang, 2013). In addition to being involved in energy production, mitochondria produce several epigenetic-related metabolites, such as NAD+, ATP, alpha-ketoglutarate and acetyl coenzyme A, which are necessary substrates for nuclear transcriptional and epigenetic processes, such as chromatin remodeling, histone modification, and nucleosome positioning (Shaughnessy et al., 2014). In the human mitochondrial genome, the methylation pattern is uniform and constant throughout the molecule, although there are differentially methylated sites between different tissues, different samples collected at different time points and at different gene start sites, suggesting the existence of a regulatory mechanism of the mtDNA methylation that needs to be further elucidated (Ghosh et al., 2014). The induction of reactive oxygen species in human cells, for example, leads to a decrease in the mtDNA methylation, probably due to the greater compactness of the mtDNA in order to protect it, since the nucleoid protein TFAM has greater DNA binding affinity with damaged DNA (Rebelo et al., 2009). Although recent studies detected histones in the mitochondria (Choi et al., 2011), the general consensus is that mitochondrial histone complexes do not exist and that the methylation of mtDNA also plays a role in its stability and functioning (Byun and Baccarelli, 2014).

Data on mtDNA methylation are focused on human samples, given the increasing evidence of mtDNA methylation role in human diseases and its potential use as a biomarker for harmful environmental and nutritional factors (Iacobazzi et al., 2013). In addition, although an increasing amount of data indicate that mitochondria are important in fungal virulence and survival (Bambach et al., 2009; Qu et al., 2012; Thomas et al., 2013), little is known about *C. albicans* mtDNA variability and its effect in pathogenicity. There is no currently available data on *C. albicans* mtDNA methylation and how host conditions may influence it.

To test the hypothesis that methylated cytosines are present in *C. albicans* mtDNA, we performed Whole-Genome Bisulfite Sequencing (WGBS) using DNA isolated from the reference strain SC5314. Yeast cells were grown on YPD plates (1% w/v yeast extract; 2% w/v peptone, 2% w/v dextrose, 2% w/v agar) and a single colony was used for overnight growth on YPD broth at 28°C, 150 rpm. Total yeast DNA was extracted from samples as described previously (Wach et al., 1994). DNA was treated with sodium bisulfite (Zymo EZ DNA Methylation Gold kit) prior to the addition of the adapters during a preparation of the libraries, according to the Illumina TruSeq Nano kit manufacturer’s instructions. Paired-end 2 bp × 300 bp runs were performed by the MiSeq Illumina method. FastQC v.0.11.4 software (Andrews, 2010) was used to evaluate sequencing quality and trimming was performed with Trim Galore 0.4.0v. Read alignment was carried out with Bismark Bisulfite Mapper 0.13.0v (Krueger and Andrews, 2011) and duplicated mapped reads removed. Alignment was performed using the reference sequence publicly available for the SC5314 strain (Assembly 22: A22-s05-m04-r02, from candidagenome.org). Interestingly, a previous analysis showed that the mtDNA of the strain L757 had only 1% sequence variability (approximately 300 nucleotides) in comparison to the SC5314 mtDNA sequence (Bartelli et al., 2013). The percent methylation was calculated with the bismark_methylation_extractor tool implemented in the Bismark program (bedGraph files) and the R package MethylKit v. 0.9.5 (Akalin et al., 2012) for the different CpG, CHH and CHG contexts. The sites analyzed were filtered and only those with Phred score > 20 and minimum 10 reads coverage were considered. Histograms with coverage distribution were checked and no duplication bias was detected. Differentially methylated cytosine calculations (Wang et al., 2011) and Pearson correlation coefficients were calculated as implemented in MethylKit v. 0.9.5 (Akalin et al., 2012). For this analysis, only sites with satisfactory quality and coverage on both samples were considered. MtDNA mappings were visualized in the Integrative Genomics Viewer v.2.3.57 (Robinson et al., 2011).

The complete mtDNA of this pathogen is 40 kb long, and at least 85% of the *C. albicans* SC5314 mtDNA sequence (34,233 bp) with an average coverage of 162x was obtained after bisulfite treatment, sequencing and assembly. Fragments of mtDNA not assembled correspond to the two repetitive and inverted regions present at the two ends of *C. albicans* mtDNA, with approximately 7 kb each (**Figure 1A**, top row). Methylation levels were analyzed in three different contexts, CpG, CHH and CHG, on both strands. This sample exhibited a global hypermethylation pattern, with a mean methylation per cytosine of 99%. In addition, the methylation distribution shows that approximately 97, 96, and 94% of the mtDNA cytosines have the methylation rate equal to or greater than 95% in the CpG, CHH, and CHG contexts, respectively. Another strain, L757, a more recent *C. albicans* pathogenic isolated from a candidemia patient (Padovan et al., 2009), was analyzed in parallel after growth under the same conditions and similar results were identified (**Figure 1B**, top row).

**FIGURE 1:**
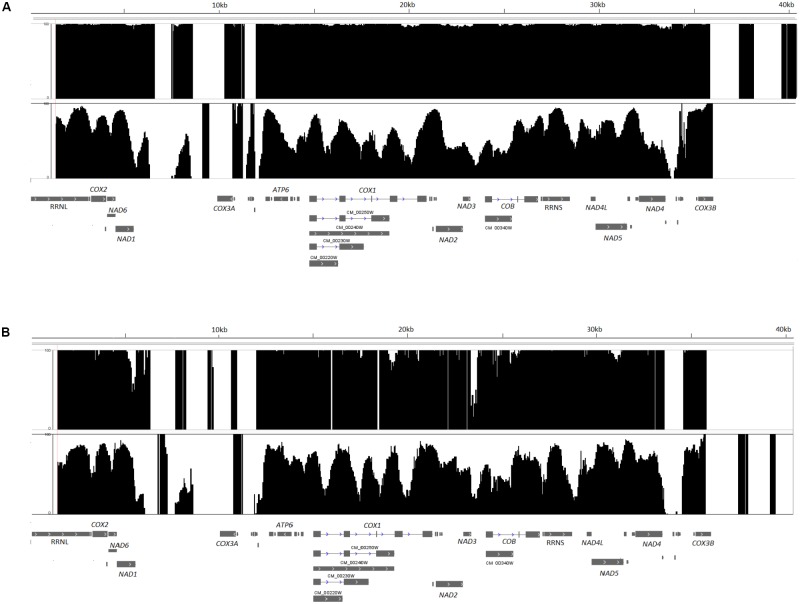
Methylation maps of *Candida albicans* mtDNA, strains SC5314 **(A)** and L757 **(B)**. The figure depicts the percentage of methylation per cytosine on CpG, CHH, and CHG contexts for SC5314 **(A)** or L757 **(B)** (top row) showing hypermethylation and SC5314-GTH12 or L757-GTH12 (bottom row) with variable methylation distribution and hypomethylated cytosines. The two large portions of the mtDNA without percentage of methylation in the figure correspond to the two repetitive inverted portions of *C. albicans* mitochondrial genome (5,540–12,382 and 33,578–40,420 bp).

To test the hypothesis that growth conditions affect the methylation profile of *C. albicans* mtDNA, strains SC5314 and L757 were analyzed after 12 weeks of continuous growth under 5–15% oxygen level (hypoxia), YPG broth (1% w/v yeast extract; 2% w/v peptone, 2% w/v glycerol) at 37°C, named SC5314-GTH12 or L757-GTH12 (Glycerol, Thirty-seven °C, Hypoxia, 12 weeks). The oxygen tension in the atmosphere is approximately 21% (O_2_ pressure of 159 mmHg at sea level), known as normoxia, and healthy tissues in the human body range from 2.5 to 9% O_2_ (Grahl et al., 2012). This lower oxygen tension (<21%), known as hypoxia, varies depending on the anatomical site or tissue inflammation (Grahl et al., 2012). When subjected to temperatures above 30°C *C. albicans* activates its heat shock response, leading to the transcription of several genes, especially chaperones, by the heat shock factor 1 (Hsf1) (Nicholls et al., 2009). While the response to heat shock and hypoxia can be related and dependent, several studies show that changes in the carbon source strongly influence *C. albicans* stress resistance, and therefore, it is essential to consider the pathogen response to combinatorial stressors rather than to individual stress factors commonly studied separately *in vitro* (Brown et al., 2014). The combinatorial stresses are relevant when representing many host niches, such as a mucosal invasion (oxidative stresses plus water balance) or a kidney infection (cell adaptation to high salt concentrations plus endogenous reactive oxygen species) (Brown et al., 2014).

We obtained at least 82% (33,151 bp) of the sequence of the mtDNA of strain SC5314-GTH12, with 119x average coverage. This sample showed reduced levels of methylation, with higher heterogeneity and greater variability in the methylation levels of the mtDNA with a mean methylation of 60%, including non-methylated sites (0%) that were not seen in the SC5314 mitochondrial methylome previously analyzed (**Figure 1A**, bottom row). The methylation distribution showed that only 14, 9.1, and 12.2% of the cytosines analyzed had values equal to or greater than 90% methylation in CpG, CHH, and CHG, respectively, values that are much lower than the ones observed for strain SC5314. Again, similar results were identified for the other *C. albicans* strain (L757-GTH12) (**Figure 1B**, bottom row) when grown under this same condition.

The variability in cytosine methylation percentages, with values ranging from 0 to 100% at each site, is related to the heterogeneity of the sample, which is composed of a mixture of *C. albicans* cells and/or the occurrence of several copies of the mtDNA per cell, which may have heterogeneous methylation profiles. Also, for some genes, within a predominantly methylated population, the occurrence of unmethylated copies between them is common. This indicates that methylases may have a limiting rate, which results in incomplete methylation or that the transition from active to inactive transcription occurs through a passive dilution of methylated copies during replication (Mishra et al., 2011).

To identify differentially methylated cytosines between samples SC5314 and SC5314-GTH12, bases with *q*-value < 0.01 and % methylation difference > 30% were considered (Wang et al., 2011; Akalin et al., 2012). When comparing SC5314 and SC5314-GTH12, we identified 5,520 differentially methylated cytosines, 530 in the CpG, 4,355 in the CHH and 635 in the CHG context, all of them hypomethylated in SC5314-GTH12 as compared to SC5314. Only 1,949 cytosines (35.3%) were located in coding regions. Among the genes with the highest number of differentially methylated cytosines between SC5314-GTH12 and SC5314, were RRNL, *COX1*, *COB* and *NAD4*, including possible endonucleases involved with *COX1* and *COB* splicing (**Table 1**). When analyzing differentially methylated cytosines between strain SC5314 and L757, we could detect genes with cytosines hypo and/or hypermethylated between SC5314 *versus* L757 and SC5314-GTH12 *versus* L757-GTH12 (**Table 1**), indicating that mtDNA methylation is likely strain specific and associated with the different adaptation patterns of *C. albicans* clinical isolates in the human body.

**Table 1:** Differentially methylated cytosines (C^diffmet^) between samples SC5314, SC5314-GTH12, L757, and L757-GTH12.

				mtDNA localization	
Samples	Methylation	Context	Total	Non-coding regions	Genes (number C^diffmet^)
SC5314 *versus* L757	Hypo	CpG	530	332	RRNL (35), tRNA-Ala (5), *COX2* (2), *NAD6* (1), *NAD1* (3), CM_00230W (20)^∗^, CM_00220W (13)^∗^, CM_00240W (35)^∗^, CM_00250W (51)^∗^, *COX1* (66), *NAD2* (7), *NAD3* (2), CM_00340W (11)^∗∗^, *COB* (29), RRNS (7), *NAD4L* (4), *NAD5* (6), *NAD4* (14), tRNA-Met (2), *ATP6* (6), *ATP8* (2), tRNA-Pro (3), tRNA-Gly (5)
	Hypo	CHH	4,355	2,860	RRNL (149), tRNA-Ala (7), *COX2* (36), *NAD6* (28), *NAD1* (28), *COX1* (505), CM_00220W (121)^∗^, CM_00230W (205)^∗^, CM_00240W (325)^∗^, CM_00250W (437)^∗^, *NAD2* (120), *NAD3* (32), *COB* (216), CM_00340W (131)^∗^, RRNS (39), *NAD4L* (21), *NAD5* (79), *NAD4* (124), tRNA-Met (6), *ATP6* (66), *ATP8* (13), tRNA-Pro (8), tRNA-Cys (9), tRNA-Gly (9)
	Hypo	CHG	635	379	RRNL (47), tRNA-Ala (1), *COX2* (6), *NAD6* (4), *NAD1* (8), *COX1* (66), CM_0022OW (11)^∗^, CM_00230W (22)^∗^, CM_00240W (39)^∗^, CM_00250W (52)^∗^, *NAD2* (20), *NAD3* (4), *COB* (37), CM_00340W (20)^∗∗^, RRNS (6), *NAD4L* (5), *NAD5* (14), *NAD4* (28), *ATP6* (3), *ATP8* (3), tRNA-Pro (2), tRNA-Gly (2)
SC5314 *versus* L757	Hypo	CHH	3	0	RRNL (1), CM_00230W (2)^∗^, CM_00220W (2)^∗^, CM_00240W (2)^∗^, CM_00250W (2)^∗^, *COX1* (2)
SC5314-GTH12 *versus* L757-GTH12	Hyper	CpG	20	11	*ATP6* (1), CM_00240W (7)^∗^, CM_00250W (7)^∗^, *COX1* (7), *NAD2* (1)
	Hypo	CpG	17	10	*ATP6* (2), *COX2* (1), *COX1* (1), *COB* (3)
	Hyper	CHH	95	70	*ATP6* (2), CM_00240W (20)^∗^, CM_00250W (20)^∗^, *COX1* (20), *NAD2* (2), *NAD4* (1)
	Hypo	CHH	92	71	*ATP6* (2), RRNL (1), *COX2* (2), CM_00220W (2)^∗^, CM_00240W (2)^∗^, CM_00230W (2)^∗^, CM_00250W (3)^∗^, *COX1* (5), *NAD2* (2), CM_00340W (6)^∗∗^, *COB* (8), *NAD4* (1)
	Hyper	CHG	13	8	CM_00250W (4)^∗^, CM_00240W (4)^∗^, *COX1* (4), *NAD2* (1)
	Hypo	CHG	6	4	*COB* (2), CM_00340W (1)^∗∗^


*Bases with q-value < 0.01 and difference of methylation greater than 30% for cytosines with a minimum coverage ≥ 10 on both samples. All C^*diff met*^ were hypomethylated on sample GTH12 as compared to their corresponding (SC5314 or L757). Endonucleases coded within *COX1* (^∗^) or *COB* (^∗∗^) gene sequences.*

We observed that the *C. albicans* methylated mtDNA cytosines were mainly distributed inside introns, exons, and intergenic regions (**Figure 1**). *C. albicans* mtDNA transcription is polycistronic, and there are only eight promoters involved in the regulation of eight transcription units (TUs) (Kolondra et al., 2015). Among them, only TU7 promoter (CTCCTTATA), controlling the expression of *NAD4L*, *NAD5* and three tRNAs genes, located between positions 29,532 and 29,540 bp, has cytosines in its sequence. Therefore, these data indicate that the methylation of mtDNA gene promoters is not a mechanism as relevant as the gene body methylation for *C. albicans* may be. For strain SC5314, cytosine methylation in this TU7 promoter was close to 98%, while the values dropped to 67% for sample SC5314-GTH12.

Using the Pearson correlation coefficient, we identified that the mtDNA methylation of cytosines between samples SC5314 and SC5314-GTH12 is not strongly correlated, with values ≤0.35 (0.35 CpG, 0.25 CHH, and 0.28 CHG), suggesting an environmental influence on the overall level of methylation of *C. albicans* mtDNA. Global methylation profiles were also analyzed by the Spearman correlation coefficient, with similar results (Bartelli, 2016).

In our samples, methylation occurred in the CpG and non-CpG (CHH and CHG) contexts, uniformly throughout the molecule, in both gene bodies and non-coding regions. In *C. albicans*, other authors (Mishra et al., 2011) observed that the methylation of nuclear genome cytosines is also distributed throughout the gene bodies, and the presence of methylation plays a direct role in the inhibition of transcription. In the human nuclear genome the methylation in the gene body is a very frequent phenomenon and CpG methylation in nuclear gene promoters may not play such a large role in the gene regulation, with histone acetylation or methylation being more relevant in this context (Maunakea et al., 2010). Increased methylation along the gene sequence is associated with increased transcription (Flanagan and Wild, 2007; Cokus et al., 2008; Rauch et al., 2009; Maunakea et al., 2010). In our samples, we observed a decrease in the overall methylation profile of the SC5314-GTH12 sample, which may be associated with the transcriptional profile of *C. albicans* in adaptation to hypoxia, which is characterized by increased expression of genes associated with glycolysis and decreased expression of genes involved in the mitochondrial tricarboxylic cycle (Krebs cycle) and oxidative phosphorylation, that are oxygen dependent (Setiadi et al., 2006; Synnott et al., 2010).

The mtDNA methylation pattern and its consequences to the cell are still poorly understood and may be associated with cellular and mitochondrial responses to environmental stressors (Shaughnessy et al., 2014). Although there are few studies, mtDNA methylation is likely to influence gene expression, biogenesis and mitochondrial functions (Iacobazzi et al., 2013). In humans, the mtDNA methyltransferase 1 (mtDNMT1) is associated with mtDNA CpG, especially in D-loop, where the origin of replication and promoters are located, and also in rRNA and protein-coding gene sequences (Shock et al., 2011). Non-methylated sites in the mtDNA are caused by proteins that hinder DNMT access and consequently, cytosine methylation (Rebelo et al., 2009). In humans, DNMTs have differential access to different mtDNA sites, based on the level of proteins in their nucleoids. The ratio between the concentrations of TFAM, the major constituent protein of human nucleoids, and mtDNA may be one of the major regulators of mitochondrial activity (e.g., replication and gene expression) and may vary according to the metabolic demand of the cell. Methylation levels may be influenced by factors known to alter nucleoid structure, such as a decreased TFAM/mtDNA ratio, which leads to a less compacted mtDNA that is more accessible to DNMT, and therefore, have higher methylation rates (Rebelo et al., 2009). Therefore, unlike the common effect expected in nuclear DNA, higher levels of methylation in mtDNA may be associated with its lower compaction and, consequently, increased gene activation. In *S. cerevisiae*, Abf2 level, which is homologous to TFAM, is known to be variable in yeast cells according to its metabolic necessity. The Abf2/mtDNA ratio is reduced under conditions that favor aerobic respiration, while their levels increase under conditions unfavorable to respiration (Xiong and Laird, 1997), suggesting an increase in mtDNA compaction under conditions unfavorable to respiration that would lead to lower methylation rates by hindering the access of DNMTs.

In our experiments we also observed that several sites remained with high methylation levels in SC5314-GTH12 or exhibited differential methylation between different strains (SC5314 and L757) (**Figure 1** and **Table 1**). This probably occurs because the distribution of DNA binding proteins, such as the human TFAM, is not constant throughout mtDNA, and some regions have a more discrete decrease in methylation even with increased concentration of this protein in the cell (Rebelo et al., 2009). In *C. albicans*, nucleoid proteins, such as Gcf1 which is the most important described so far, participate in several functions, such as mtDNA replication (Visacka et al., 2009) and may have a role in the regulation of methylation of specific sites in the mtDNA to which they are associated and of the level of compactness they may induce to the molecule. When analyzing the Pearson correlation coefficients between global methylation profiles of samples SC5314 and SC5314-GTH12, we observed medium to low values of correlation and occurrence of differentially methylated sites between different culture conditions and strains. These results indicate that in addition to environmental conditions affecting the mtDNA methylation patterns, this response could be lineage-specific and related to adaptation and differential virulence of these strains, as previously described by our group (Padovan et al., 2009).

## Accession Numbers

Raw sequencing data are available from the NIH SRA (BioProject PRJNA395439), under BioSample Accession Numbers SAMN07406734 (SC5314), SAMN07406735 (SC5314-GTH12), SAMN07406736 (L757), and SAMN07406737 (L757-GTH12).

## Author Contributions

TB: planned and performed the experiments, analyzed the data, discussed the results and implications, performed the sequencing and bioinformatics analysis, and wrote the manuscript. DB: discussed the results and implications and edited the manuscript. MB: planned and supervised the experiments, data analysis, obtained funding, and edited the manuscript.

## Conflict of Interest Statement

The authors declare that the research was conducted in the absence of any commercial or financial relationships that could be construed as a potential conflict of interest.
